# Understanding the role of peer pressure on engineering students' learning behavior: A TPB perspective

**DOI:** 10.3389/fpubh.2022.1069384

**Published:** 2023-01-06

**Authors:** Lin Xu, Jingxiao Zhang, Yiying Ding, Junwei Zheng, Gangzhu Sun, Wei Zhang, Simon P. Philbin

**Affiliations:** ^1^School of Foreign Languages, Northwest University, Xi'an, China; ^2^School of Economics and Management, Chang'an University, Xi'an, China; ^3^Faculty of Civil Engineering and Mechanics, Kunming University of Science and Technology, Kunming, China; ^4^School of Civil Engineering, Zhengzhou University, Zhengzhou, Henan, China; ^5^Institute of China's Science Technology and Education Policy, Zhejiang University, Hangzhou, China; ^6^School of Engineering, London South Bank University, London, United Kingdom

**Keywords:** peer pressure, peer influence, theory of planned behavior, engineering students, learning intention, learning behavior

## Abstract

**Introduction:**

With the advent of the digital age, the gradually increasing demands of the engineering job market make it inevitable that engineering students face the pressures that arise from academic life with their peers. To address this issue, this study aims to explore the influence of engineering students' peer pressure on learning behavior based on the theory of planned behavior (TPB).

**Methods:**

In addition to attitudes, subjective norms, and perceived behavioral controls inherent in TPB, two new dimensions—gender difference and peer academic ability—were incorporated to construct a framework of the dimensions of peer pressure as affecting engineering students as well as an expanded model of TPB. A questionnaire survey was conducted with 160 college engineering students and a structural equation model (SEM) was used to test the hypotheses.

**Results:**

The result showed that positive peer pressure can increase engineering students' learning intention and thus promote learning behavior. It was also determined that the TPB model can effectively explain the effect of peer pressure on learning behavior, in addition to expanding and reshaping the relationship between the attitudinal dimension in the TPB model.

**Discussion:**

From the results, it is clear that positive attitudes toward learning can trigger positive peer pressure. Good group norms can induce peer pressure through rewards and punishments as a way to motivate students' learning intention and learning behaviors. When peer pressure is perceived, students mobilize positive emotions toward learning. Meanwhile, both male and female engineering students are also significantly motivated by high peer achievement, and high-performing female students motivate their male peers, which leads to higher graduation rates.

## Highlights

- Innovatively explored the role of peer pressure in influencing engineering students' learning behavior by applying the theory of planned behavior (TPB).
- Empirically proposed a hypothetical model as an influence mechanism through constructing a basic framework of five peer pressure dimensions based on TPB.
- Demonstrated that positive peer pressure can increase engineering students' learning intention and thus promote learning behaviors, and the TPB model can effectively explain the effect of peer pressure on learning behavior.
- Extended the application of TPB, expanded and reshaped the relationship of attitudes in TPB, and enhanced the explanatory power of the model.
- Proved that the result has certain practical and theoretical implications that further enriches the theoretical knowledge base on engineering education and broadens our understanding of peer pressure, and its implications for enhancing engineering students' learning abilities.

## 1. Introduction

With the advent of the digital era and the corresponding urgent need for digital transformation, the engineering job market increasingly demands international engineering students equipped with a comprehensive set of skills. Driven by digital technology, the increasingly complicated and challenging work environment has enhanced the requirements for engineering graduates in terms of academic achievement and wider skills. This includes not only professional learning achievement, but also comprehensive skills and abilities (1). Indeed, the rapid development of science and technology in society requires engineering students to have the capacity for lifelong learning. Furthermore, students engaged in an application-oriented major in engineering need to keep abreast with the times, leverage innovation in their traditional learning methods, and actively use emerging information technology (IT) to enhance their problem-solving skills (2). It is not only necessary to have a solid grasp of professional knowledge and proficiency in engineering technologies required by the curriculum, but also to continuously expand interdisciplinary knowledge as well as learn emerging IT applications. Driven by the internet and various digital technologies, such as cloud computing, artificial intelligence and big data, engineering education has become integrated with a range of different digital technologies. Simultaneously, teaching methods are diversifying and developing, and the ways of learning for engineering students are changing dramatically (3). Instead of a single teacher-led traditional teaching style, students are guided to achieve improved learning objectives through independent as well as collaborative peer learning.

Under such circumstances, engineering students inevitably face many psychological stresses, the most concerning of which is the stress generated by interacting with peers in collaborative learning environments. Peer pressure describes the sense of imbalance and psychological conflict that arises in comparison with the peers around students or the peer group the students are in. The influence of peer groups, as informal groups, on individuals is largely realized in the form of peer pressure (4). Furthermore, Clasen and Brown (5) defined peer pressure as the psychological impact of peer expectations on the attitudes, lifestyles and behaviors of individuals. Adults feel peer pressure when adopting goals, beliefs, and behaviors shared by their peers. Peer pressure also exists as a social effect in higher education, affecting the academic performance of students from secondary education through to higher education levels (6). Positive peer pressure motivates individual students to remain aligned with their class group, thus maintaining the common development of the peer group and ensuring the achievement of shared goals. The learning outcomes of students are often positively correlated with the performance of their peers because they can learn from each other (7). A learning environment with peer pressure can also affect the overall achievement of individuals and influences their motivational beliefs, attitudes toward learning, and expectations of success.

Many courses for engineering majors insist upon the inclusion of peer collaboration by students in order to achieve academic goals (8). For example, the core of a particular BIM (Building Information Modeling) course is effective collaboration, which guides students to share values and beliefs in the classroom and establishes common learning goals through communication (3). Moreover, intensive information exchange with classmates under collaborative circumstances can easily causes peer pressure. This is human nature (9). Positive peer pressure affects students' learning by pushing them to make their attitudes, beliefs and behaviors conform to the group norms. Students take the excellent peers around them as a benchmark and imitate their behavior when cooperating with them (10). Peer pressure is an external factor that drives BIM learning and engineering practice, and a growing body of research highlights the value of peer pressure to the learning process and the benefits of its implementation as an important pedagogical approach to increasing student motivation.

The theory of planned behavior (TPB) is a theory that explains and predicts individual behavior, concerning behavioral intention as a direct determinant of behavior. The theory has been shown to have effective explanatory power and is used in a variety of fields. Students' learning behavior is essentially determined by the learning intention, while TPB explains and predicts their behavior from the perspective of the relationship between intention and behavior (11). In view of this assertion, the present study adopts TPB as a guiding framework to explore how peer pressure affects engineering students' learning intention, and thus their corresponding learning behavior.

Consequently, this empirical study focuses on addressing the following questions: (1) What influence does peer pressure exert on engineering students' academic behavior? (2) Also, how does the process of peer pressure operate? The study herein categorizes peer pressure according to attitude, subjective norm, perceived behavioral control, gender difference, and peer academic level based on TPB. The study hypothesizes that the attitude dimension has a significant direct influence on perceived behavioral control and subjective norm, and gender difference and peer academic ability affect learning behavior through perceived behavioral control, subjective norm and learning intention. From the theoretical perspective for engineering education, the implication of the study is that the TPB model can explain the effects of peer pressure on the learning behaviors of engineering students and the study expands the traditional TPB model through inclusion of the attitude relationship. In addition, understanding and mastering the forms and mechanisms of peer pressure and its effects are of high theoretical value as well as having further implications for research on engineering education in the digital era. From the practitioner perspective, the research study is instructive for engineering educators to apply appropriate peer pressure, especially the use of reward and penalty-oriented structures to improve engineering students' academic performance and overall competencies. Consequently, engineering students are better motivated by peer pressure to improve their personal learning outcomes and academic performance.

## 2. Literature review

### 2.1. Theory of planned behavior

**Figure 1:**
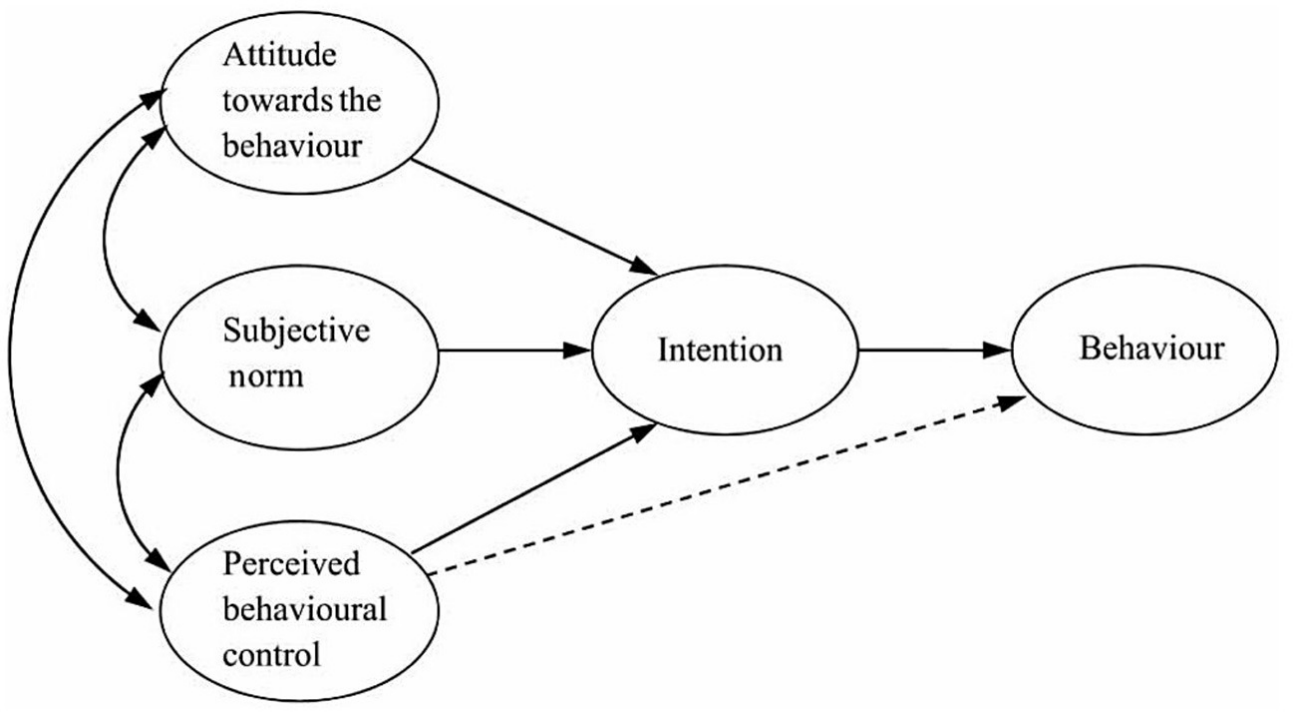
Ajzen's theory of planned behavior (TPB) (13).

The Theory of Planned Behavior (TPB) possesses significant explanatory power and authority in studying the mechanism of behavioral intention. TPB integrates five elements, namely: attitude, subjective norm, perceived behavioral control, intention and behavior, which comprehensively explains the emergence of multiple behaviors (12). As shown in Figure 1, behavior intention is a direct determinant of behavior, thereby indicating that people are willing to exert the effort to try or perform a particular behavior (13).

#### 2.1.1. Attitude

Attitude is an individual's overall evaluation of the implementation of a particular behavior (14). Psychologists believe that attitudes are formed gradually through interactions with others and the surrounding environment, i.e., attitudes have social properties (15). As a central factor influencing behavioral intentions, attitudes also interact with subjective norms and perceived behavioral control, but the specific mechanisms vary depending on the subject and the context in which the study is conducted (13). Indeed, the TPB theoretical framework suggests that attitudes may also inversely influence subjective norms and perceived behavioral control, and the expansion and reshaping of attitudinal relationships can enhance the explanatory degree of the model. This study concludes that the more positive students' attitudes toward learning and peer pressure, the more they receive positive personal norms and exemplary normative pressure, which affects learning behavior.

#### 2.1.2. Perceived behavioral control

Perceived behavioral control concerns the individual's perception of the ease of performing a specific behavior. An individual's subjective self-perception of whether he or she can perform a particular behavior includes their perceived ability, beliefs, and confidence to enact the behavior. Furthermore, it is much more likely for an individual to develop the intention to perform a behavior when being aware of having stronger inner ability and perceiving that there are more resources and opportunities available and fewer obstacles externally. In this context, the perceived behavioral control over the behavioral intention is stronger (16).

#### 2.1.3. Subjective norm

Subjective norm pertains to the social pressures that individuals perceive regarding whether or not to enact a specific behavior. In particular, the attitudes and actions of significant others toward a particular behavior are an important source of the subjective norm. Ackerman and Gross (17) showed that the rules and learning environment within the classroom have an important impact on academic performance. The subjective norm is structurally composed of personal norms, exemplary norms, and directive norms. Personal norms are equivalent to self-identity or moral norms. Directive norms refer to certain behaviors that are expected of an individual by those around him or her. Exemplary norms mainly refer to the social pressure felt by the individual. Rivis and Sheeran (18) concluded that directive norms have more limited predictive power on behavior than exemplary norms. Therefore, this study focuses on personal norms vs. exemplary norms at the subjective norm to analyze the effects of peer pressure on the learning behaviors of engineering students.

In summary, TPB can be a good predictor of effective learning behavior among students. As a wellknown theoretical model for predicting and explaining behaviors, TPB has been widely applied to many areas of human life, such as leisure choices, the job search behaviors of college graduates, and health-related behaviors (19). Many scholars have used TPB to construct models based on different perspectives to study the factors influencing various behavior intentions. However, after reviewing the extant literature, it has been found that TPB has not been applied to study the influence of peer pressure on the learning behavior of engineering students. As the authoritative theory of behavioral research, TPB provides a suitable research perspective and theoretical basis for this study. Therefore, this study utilizes the TPB framework to investigate the factors and mechanisms of peer pressure on the learning behavior of engineering students.

### 2.2. The connotations of peer pressure

Peer pressure has become a hot topic in recent years in the study of individual psychological dispositions. Peer pressure can be defined as the experience of stress that arises when one gives up one's self to conform to the choices of one's peers for fear of being ostracized (20). As Haun and Tomasello (21) identified, there exists an invisible force created by the opinions of a peer group that causes each member to consciously or unconsciously align with the majority and thus change attitudes, values, and behaviors to fit the group norms. This invisible influence is called peer pressure. In this regard, individuals interact with peer groups to perceive peer pressure, which results in beliefs and behaviors that conform to the group's requirements, i.e., conform to group norms. Although peer pressure is not compulsory as a subjective feeling, it is an irresistible force for individuals, and they will therefore behave in a herd-like manner to satisfy the public by becoming oblivious to which behavior is right and which is wrong (22).

Peer pressure is a multidimensional construct varying in strength and direction across different behaviors. Clasen and Brown (5) classified peer pressure into five different domains: peer involvement, inappropriate behavior, peer conformity, participation in school activities, and participation in family activities. In 1993, Mansiki (23) suggested that the effects of peer pressure are influenced by factors internal to the students themselves and external to the learning environment. Internal factors refer to demographic heterogeneity, namely gender, race, academic ability, attitudes, motivation, and expectations (24). External factors refer to the school and home learning environment, including teachers, teammates, teaching strategies, family preferences, and subjects of study (25). The scholars Barron and Gjerde (26) and Lazear and Shaw (27) divided peer pressure into internal peer pressure and external peer pressure. Among them, internal peer pressure arises from internal psychological feelings, specifically jealousy and guilt, while external peer pressure arises from peer punishment, i.e., the penalty to be paid.

### 2.3. Connotation of the peer pressure dimension from the perspective of TPB

**Figure 2:**
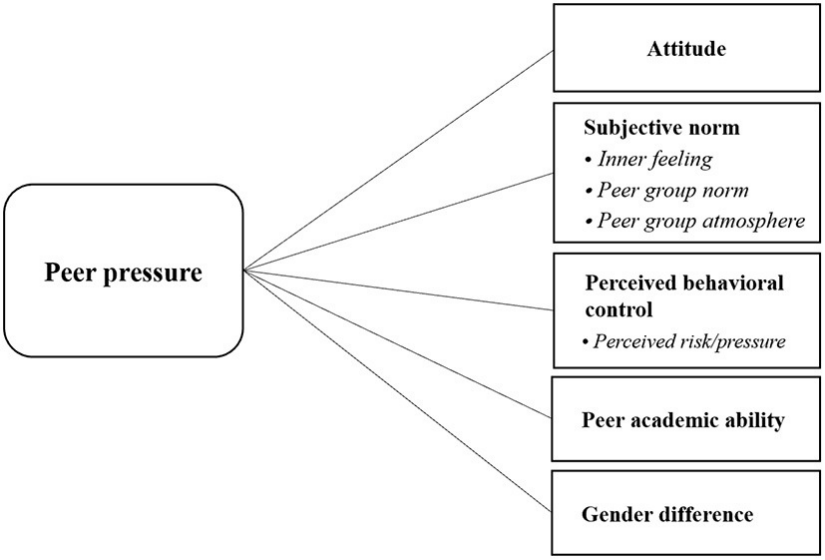
Connotation of peer pressure dimension.

Peer pressure is not a unitary (i.e., one-dimensional) construct, but rather a multidimensional one (28). Based on the above dimensions of TPB and the specific connotation of peer pressure, the relevant literature was further sifted and summarized, and peer pressure was categorized into three dimensions: attitude (in this study, attitude refers to engineering students' attitudes toward learning and peer pressure), subjective norms and perceived behavioral control (23, 26), as shown in Figure 2. Among them, the connotation of subjective norms includes group atmosphere, group norms and inner feeling, and perceived behavioral control includes perceived pressure. In addition to the above three dimensions, for enhancing the explanatory power of TPB, two important factors inherent in peer pressure—gender difference and peer academic ability—were added to this study based on the analysis of existing studies in the literature.

#### 2.3.1. Subjective norm

##### 2.3.1.1. Inner feeling

According to Barron and Gjerde (26), individual psychological feelings are a major influence on peer group stress, specifically jealousy and guilt. Kandel and Lazear (22) also identified feelings of shame as an influencing factor of peer pressure. These feelings are associated with an individual's self-esteem. Khampirat (29) suggested that self-esteem plays an important role in student development and the concept of self-esteem refers to an individual's judgment of self-worth and is associated with learning outcomes.

##### 2.3.1.2. Peer group norms

External peer pressure stems from fear of punishment among peer groups. Once peer group norms are established, they exert pressure on individuals within the group, and deviations from the norms may be met with praise, shame, and rejection. Based on such group peer pressure, changes can be affected in individual knowledge and values (30). Rand et al. (31) showed that people have a strong need to assess their own views and values by comparing them with those of others and that there is a strong tendency for such comparisons to be consistent. Moreover, people within a peer group can reward or punish the behavior of its members, and people expect uniform standards of behavior (32). Pitt et al. (33) also noted that increasing peer awareness of academic productivity and bonuses can serve as effective motivational models to increase positive peer pressure.

##### 2.3.1.3. Peer group atmosphere

Group atmosphere refers to the learning environment in which peers communicate and interact with each other. In a good interactive environment, peer groups are positively enthusiastic about learning and are willing to share their learning experiences and achievements with each other (33, 34). In such an atmosphere, students who are unwilling to learn are ostracized and reprimanded by their peers, and based on this positive peer pressure, students maintain orderly and efficient study habits among the group, thereby creating a strong academic atmosphere (35).

#### 2.3.2. Perceived behavioral control

##### 2.3.2.1. Perceived risk/pressure

Risk perception refers to the subjective judgments made by individuals about the characteristics of a certain behavior. When the perceived risk brings positive utility, individuals choose to perform the behavior. When the perceived risk is a negative utility, the individual will stop the behavior. The same is true for the perception of stress, for example, Herzenstein et al. (36) considered the difficulties encountered in the learning process as part of the perceived risk. Whereas Nielsen (37) introduced the concept of “learning uncertainty,” which means that when individuals have one more thing to learn, they also have one more chance to make a mistake. This shows that the learning process is risky and stressful. When individuals perceive greater risks, they experience greater negative peer pressure and are more likely to become resistant learning, which in turn affects their learning behaviors.

#### 2.3.3. Other inherent factors

##### 2.3.3.1. Gender difference

Gender has been identified as a significant factor influencing the effects of peer pressure on university students (4, 38). Ficano (4) found that male college students are significantly motivated by their same-gender peers who achieve high academic success. Whereas Hill (39) emphasized that female peers who excel academically motivate their male peers and enhance their interest in learning, thereby increasing graduation rates. The study also found that male students appear to be more influenced by their peers in terms of academic ability than female students, and they appear to be influenced by different levels of male and female student peers (40). Griffith and Rask (24) attempted to examine differences in sensitivity to peers across groups, focusing mainly on gender. Although there is no clear consensus, male students appear to be more sensitive to peer competence than female students.

##### 2.3.3.2. Peer academic ability

Many studies from the literature have found that peers with high academic ability create positive peer pressure on other students, which in turn has a positive impact on their learning outcomes (10, 41). According to the work of Booij et al. (41), most peers are happy to help influence others regardless of whether they are academically low, medium, or high level. In higher education, since peers take the same or similar courses at university, they interact in the classroom through discussion and collaboration. In this context, some students of high academic ability invariably teach their peers specific study skills they possess or share their own good study habits such as effective schedule-keeping, which allows students in that group to secure higher academic performance (10, 41).

## 3. Hypotheses and framework

### 3.1. The relationship between attitudes in peer pressure and the learning behavior of engineering students

In today's multidisciplinary society, engineering students are no longer limited to mastering a single engineering discipline. Such students need to be equipped to apply a range of knowledge and professional skills simultaneously to respond to complex engineering problems that are constantly changing (42). Studies have found that different attitudes toward learning in science, technology, engineering, and mathematics (STEM) classrooms lead to different thinking styles, learning self-efficacy, and problem-solving strategy choices (43). Therefore, attitude is one of the most important factors in the learning environment for the engineering education process. The learning ability of an individual can be increased by improving the attitude of such an individual, since attitude can influence the outcome of the student learning process (44, 45). Indeed, both positive and negative attitudes have a significant impact on the learning process and outcomes of engineering students (43, 44). Students' positive attitudes toward learning are characterized by their curiosity about engineering expertise, which are often expressed in engineering technology classrooms that require collaborative learning. They are eager to learn more about the field through collaborative interaction with their peers, and the team learning atmosphere and pressure can lead to more productive learning (46). Similarly, students with positive attitudes tend to quickly perceive positive pressure from their surroundings when confronted with difficult or boring engineering expertise, and thus perform better and faster in their learning tasks. Positive attitudes contribute to the achievement of desired learning outcomes, while negative attitudes tend to impart a resistance of teaching and learning, which affects the acquisition of knowledge and competence in specific learning areas (47).

For example, engineering drawings are considered to be an effective way to engage learners in thinking about the engineering design process and complex structures (48). Engineering drawing is one of the fundamental skills needed by all engineers to be creative and productive in the engineering profession (49). Students in all engineering disciplines need to learn engineering drafting, not only to help develop their spatial abilities, design skills and the ability to solve problems prevalent in the engineering major, but also to enable efficient communication between all professionals involved in the design and production process. In combination, these skills stimulate a positive attitude toward engineering expertise among learners (48). Researchers have noted that peer discussions and group brainstorming in engineering classrooms help foster a positive learning community and that learners' attitudes toward peer collaboration leads to different learning outcomes (50, 51). Furthermore, scholars have found that students in engineering drafting classrooms breed negative attitudes toward learning this professional skill and may inhibit cooperation with peers, which affects learning outcomes (52).

In summary, when engineering students hold positive attitudes toward learning behaviors, they are willing to engage in peer learning groups; they are able to perceive more positive peer pressure and less negative peer pressure; and thus students tend to engage in positive learning states that motivate them to achieve higher levels of academic success. Therefore, the following research hypotheses are proposed:

H1a: Attitude has a positive impact on subjective norms.
H1b: Attitude has a positive impact on perceived behavioral control.
H1c: Attitude positively influences engineering students' learning intentions through perceived behavioral control.
H1d: Attitude positively influences engineering students' learning behavior through perceived behavioral control and learning intention.

### 3.2. The relationship between subjective norm and learning intention among engineering students

The importance of collaboration in engineering has been noted in many studies, such as: “*In the new century, engineering work will increasingly involve interdisciplinary teams, globally diverse team members working together*” (53). Indeed, scientists and engineers tend to work in teams and rarely as independent workers. The collaborative nature of scientific and engineering work can be fostered through frequent group activities in the classroom (54). Team-based learning has become an important pedagogical approach in modern engineering education, where students share responsibility for each other's learning and promote deeper learning (55). In this model, group norms are invisibly formed in engineering student groups and peer pressure is induced through rewards and punishments as a way to stimulate students' learning intentions and behaviors. The disadvantage of altruistic punishment (or penalties) as a stimulus for peer cooperation is that it may interfere with students' rational cooperation (56). Moreover, Rand et al. (31) confirmed that rewards can stimulate similar peer pressure and have a greater impact in collaboration. This is consistent with the findings of Zhao (57), which found that students who received appreciation for actively contributing ideas in group discussions were rewarded with group norms and their motivation to learn was high. In contrast, teams that received penalties in the form of score deductions and were afraid of lagging behind other students' groups scored progressively higher in subsequent discussions. Under the pressure of peer group norms, students should attach importance to the success of the group, urge themselves to be responsible for their studies, develop critical and analytical thinking ability through mutual encouragement and prodding with their peers, improve their higher-order thinking ability, and ultimately achieve their learning goals.

Engineering students who do not conform to group norms suffer from internal peer pressure formed by psychological guilt, as well as internal shame brought about by peer condemnation and teacher punishment, and this shame can touch students' self-esteem, which is a key influence on engineering students' learning. Shopova (58) reported that developing engineering students' self-esteem is essential to stimulate learning intentions, thereby increasing the effectiveness and efficiency of the learning process, and improving students' ability to work in the dynamically changing engineering labor market.

At the same time, group atmosphere is an important factor in the subjective norms that influence the intention to learn. As the classroom is a basic organizational unit of school education„ the influence of the classroom atmosphere on individual students cannot be underestimated. Furthermore, engineering students' professional skills can be further increased in the presence of a good collaborative learning atmosphere rather than in a competitive or individual situation (59). In a study of engineering students' classroom emotions, Kellam et al. (60) found that throughout their studies, engineering students had the most positive learning emotions in a challenging, trusting, and positive classroom atmosphere. Many studies have shown that cooperative learning improves engineering students' academic performance to a greater extent than traditional individual learning, and that a positive peer atmosphere plays an important role in developing engineering skills learning plans, reaching agreements, project division, and cooperation (61). In addition, a cooperative learning atmosphere motivates engineering students to spend more time on learning various engineering expertise and employability skills (62, 63). In such an atmosphere, those who procrastinate on their study tasks and are unwilling to carry out their studies and will be reprimanded and ostracized by their peers, and based on this positive peer pressure, engineering students maintain a proactive attitude toward learning and engage in their studies, thereby creating a strong learning atmosphere (64).

In summary, a positive and encouraging peer atmosphere has a positive impact on students' emotions and learning intentions through the stimulation of learning situations (65, 66). Based on the above discussion, the following research hypothesis is proposed:

H2: Subjective norm has a positive impact on engineering students' learning intention.

### 3.3. The relationship between perceived behavioral control and engineering students' learning behavior

The perceptions of students of their academic self-worth are of high importance in an educational context (67, 68). Personal perceptions of the learning environment are closely related to the implementation of learning behaviors, and self-assessment of the ability to achieve goals is also an important factor in determining how individuals achieve their goals (69). In academic contexts, there is a significant positive correlation between self-perceived competence and academic achievement (70). Studies have shown that students with higher self-perceived competence for learning perform better academically, regardless of age, gender, field, discipline, and country. From this perspective, improving self-perceptions in STEM fields is particularly important for students' learning because STEM majors often have difficult introductory courses (71). There is a strong correlation between self-perception and persistence as well as academic achievement in a variety of disciplines in the engineering field (72). Many studies have identified that engineering students are more motivated to learn when they are faced with learning new engineering knowledge or skills, when they have a high self-perception of the difficulty of completion, and when they have a strong sense of control over the learning atmosphere and peer pressure (73). Students with high perceived competence tend to have high internal control autonomy and are subject to more acute peer pressure, and when urged by positive peer pressure, students will feel less inclined to procrastinate and increase their motivation to learn.

Based on a review of existing research, Stump et al. (74) concluded that perceptions strongly influence engineering students' learning behaviors. The findings also suggest that students who are confident in their ability to master course material are more willing to discuss or share their new knowledge with others. Those who are confident in their own learning abilities are more comfortable interacting with peer groups and persevere under positive peer pressure to improve their engineering expertise. They proactively seek help from higher academic peers, when necessary, receive useful feedback from other students and work together to help each other understand specialized material, improve engineering skills and perceptions of task completion.

Based on the above discussion, the following hypotheses are proposed:

H3a: Perceived behavioral control has a positive effect on engineering students' learning intention.
H3b: Perceived behavioral control has a positive effect on engineering students' learning behavior.

### 3.4. The relationship between peer academic ability and engineering students' learning behavior

High level peers contribute positively to the learning outcomes of others (7). Foster et al. (7) found that students' learning outcomes were positively correlated with the performance of their peers because they could learn from their peers. There may be a knowledge asymmetry in the academic level of engineering students, and there has been extensive research demonstrating that the academic level of less able students is aided and influenced by the peers of high academic ability around them (74). When high academic level engineering students maintain positive student interactions with less able peers, they both perceive positive peer pressure from them and develop a higher academic self-concept (67). As the more academically competent student explains concepts to his/her peers, he/she also gains a clearer, more organized understanding as a helper, deepening his/her mastery of obscure engineering concepts as well as complex engineering skills (75). In the same situation, the student being helped benefits from the opportunity to assimilate new information into his/her knowledge structure. Likewise, when students with the same level of knowledge interact with each other, cooperation brings benefits to both parties.

Consequently, it can be observed that a strong learning environment develops within the team, and students actively engage in the act of exchanging statements with each other, working together to build new understandings of material that neither knows. Whether students are at a low, medium or high academic level, most of their peers are willing to help influence others (41). This is because in the peer interaction, gaps in knowledge can be identified, which in addition to bringing about a feeling of positive learning pressure to oneself, also stimulates the refinement of knowledge and thus contributes to the improvement of one's cognition. Engineering students of different levels are involved in this cognitive process, which can be a stimulus for peer pressure. Students benefit from the enrichment process by identifying gaps in their knowledge through discussion, reflection, and mutual feedback on learning outcomes, questioning or elaborating on each other's views, and offer relevant explanations or solutions.

Based on the above discussion, the following hypotheses are proposed:

H4a: Peer academic ability has an effect on learning behavior through subjective norm and learning intention.
H4b: Peer academic ability positively influences learning behavior through perceived behavioral control.
H4c: Peer academic ability positively influences learning behavior through perceived behavioral control and learning intention.

### 3.5. The relationship between gender difference and engineering students' learning behaviors

Gender difference in peer pressure among engineering students has been identified as an important factor affecting academic performance (30, 76). Both male and female students experience different levels of pressure from peers of the opposite or same gender that can affect their academic performance in engineering courses (38). Ficano (4) found that male college students are significantly motivated by the high achievement of their peers. Male students appear to be more influenced by their peers than female students, and male students appear to be influenced differently by the academic quality of their male and female peers (77). In STEM fields, where males remain in the majority, the male-to-female ratio of engineering students makes males more likely to care about the perceptions of female students in their peer groups, and high-performing female students motivate their male peers to improve their academic skills, thereby leading to higher graduation rates (4, 39). Some studies have shown that female engineering students perform better than male students in mathematical and language-related subjects (78). In the process of collaborative peer learning, male students are more likely to be influenced by demonstrated talent by females in language or mathematics and to engage in passive or active behaviors to improve themselves. In particular, language skills are becoming increasingly important in engineering in the digital age, and the overall improvement of language skills in engineering peer groups can influence students' academic achievement.

In summary, the following research hypotheses are proposed:

H5a: Gender difference positively influences learning behavior through subjective norm and learning intention.
H5b: Gender difference positively influences learning behavior through perceived behavioral control and learning intention.

**Figure 3:**
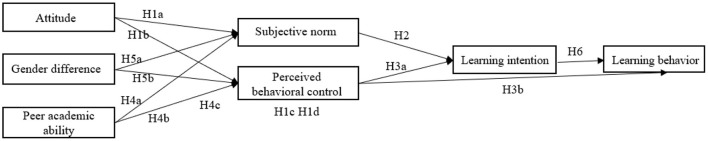
Conceptual model of PP-LB impact mechanism.

Based on the above analysis and application of the TPB model, a final hypothesis is proposed in this study: H6: Learning intention has a positive effect on learning behavior. Therefore, the conceptual model for the effect of peer pressure on engineering students' learning behavior (PP-LB) is constructed for this research study and is shown in Figure 3.

## 4. Methods

### 4.1. Data collection and participants

The questionnaire method is characterized by lower data collection costs, higher feasibility and higher external validity. In terms of collection cost, a well-constructed questionnaire can be concise and takes minimal time to complete. At the same time, questionnaires can recover a large amount of data in a short period of time, thus reducing the data collection cost. Therefore, this study uses the questionnaire method for data collection. Ajzen (14) noted that because of the highly idiosyncratic nature of behavior, there is no standard and universal TPB questionnaire, but rather one that is tailored to the nature of the specific behavior being explored and how each variable is measured. In order to further understand the influence of peer pressure on engineering students' learning intentions based on TPB, and according to Ajzen's suggestion, this study designed each variable based on the findings from existing literature and relevant established scales used in selected journals in China and internationally, or established scales developed by scholars with high citation rates. In accordance with the actual situation and specific research questions, the research team designed the entries of each variable according to the principle of simplicity and accuracy. Leading academic researchers in the field of engineering and education were also consulted to appropriately modify the measurement questions of the variables to ensure the reliability and validity of the measurement items. Finally, the language was embellished to facilitate the understanding of the survey respondents.

The survey was designed and conducted between August 3 and August 10, 2022, with a sample of participants recruited from different engineering disciplines and grades. The questionnaire was distributed using Sojump (http://www.sojump.com), a popular online survey platform in China, either by means of WeChat QR codes or links, and a total of 230 questionnaires were returned. After matching and sorting, excluding invalid questionnaires, we finally obtained 160 valid matching questionnaire sample data, with a valid response rate of 70%. The questionnaires were answered anonymously, and the participants were informed that the questionnaires were only used for academic research, and their personal privacy and security were guaranteed to be effectively safeguarded, so as to receive as many true and valid responses as possible. Data was analyzed using SPSS24.0 and structural equation modeling (SEM) in MPLUS.

As shown in Table 1, the participants consisted of 80 males and 80 females aged from 18 to 35, with a mean age of 21.41 years (SD = 3.45); 8.12% had a doctorate, 25.63% were master's students, and 66.25% had a bachelor's degree, which reflected that the participants in this survey had a high level of education. The samples are quite representative of the whole student population in terms of the distribution of degree. Participants range from six engineering majors and three universities: Xi'an Jiaotong University, Chang'an University and Northwest University.

**Table 1:** Profile of participants.

**Demographic variable**	**All (*N* = 160)**
**Frequency (%)**	
**Gender**	
Male	80 (50.00)
Female	80 (50.00)
**Age (Mean** **=** **21.41, SD** **=** **3.45)**	
18–20	86 (53.75)
21–25	58 (36.25)
26–35	16 (10.00)
**Education**	
Bachelors	106 (66.25)
Masters	41 (25.63)
Doctors	13 (8.12)
**Major**	
Civil engineering	37 (23.12)
Mechanical engineering	29 (18.13)
Geological engineering	33 (20.62)
Software engineering	24 (15.00)
Hydrology and water resources engineering	25 (15.63)
Computer science and technology	12 (7.50)

### 4.2. Measurement

The questionnaire includes three main parts. The first part gave initial information about the purpose of the questionnaire and the confidentiality of the study results. The second part mainly investigated the demographic information. The third part mainly investigated the effect of peer pressure on engineering students' learning behaviors according to 25 variables. All variables were scored on a 5-point Likert scale, where 1 means “strongly disagree” and 5 means “strongly agree”. The structure of the questionnaire and the source of the questions are shown in Appendix 1 in Supplementary material.

### 4.3. Data analysis

#### 4.3.1. Treatment of data

To ensure the quality of the data before starting the data analysis, all completed questionnaires were checked for completeness and more than 5% of missing items, as recommended by Seo (79). All questionnaires were answered completely. After matching and sorting, 70 invalid questionnaires were excluded, for such reasons as eliminating invalid questionnaires with relatively consistent responses to question items, or questionnaires with contradictory answers, resulting in 160 validly matched questionnaire sample data, with a valid response rate of 70%. As suggested by Seo (79), each item was coded as favorable or unfavorable. For items scoring less than 3, it represents unfavorable, otherwise, it means favorable. For gender, the value 1 indicated that the participant identified as male, and 2 indicated the participant identified as female.

#### 4.3.2. Statistical analysis

The structural equation model (SEM) program was used to analyze the data, and MPLUS software was used to construct SEM analysis of PP-LB impact mechanism. Different types of overall fit indices were used to evaluate the SEM models in this study, including *x*^2^/degrees of freedom ratio (*x*^2^/df), Comparative Fit Index (CFI), Tucker-Lewis Index (TLI), and Standardized Root Mean Square Residual (SRMR). In addition, the absolute fit index is often used to determine the “poor fit”, where *x*^2^*/df* is the commonly used absolute fit index. If the ratio of *x*^2^*/df* is <5, the model is considered acceptable (80). Incremental fit indices typically assess “goodness of fit”, with larger values indicating a better fit between the hypothesis model and the data. Commonly used incremental fit indices include Bentler and Bonett's Normative Fit Index (NFI), Comparative Fit Index (CFI), Tucker-Lewis Index (TLI), and Incremental Fit Index (IFI), of which both CFI and TLI are used in this study. A CFI value >0.95 (ranging from 0.00 to 1.00) represents a good fit model. A TLI value >0.9 indicates a good fit (81, 82).

## 5. Results

### 5.1. Descriptive statistics and correlation analysis

The correlations between all dimensions are shown in Appendix 2 in Supplementary material, which highlights that all variables are significantly correlated (*p* < 0.001) and none of the correlation values exceed the threshold of 0.9. This indicates that there is no multicollinearity between items (83). In addition, Fornell and Larcker (84) showed that the square root of AVE has to be greater than the correlation of other dimensions to demonstrate good discriminant validity of the scale. The findings in Appendix 2 in Supplementary material illustrate the discriminant validity of the seven dimensions, as the lowest square root of the AVE was 0.807, which was larger than the largest correlation coefficients between variables in the model.

### 5.2. EFA

Cronbach's alpha coefficient is able to test the reliability of the variables, with α > 0.6 indicating the validity of the scale, and the larger the coefficient the better the reliability of the scale (84). In this study, the reliability test was conducted using SPSS software, and as seen in Appendix 3 in Supplementary material, the α values of all variables were >0.7, which had good internal consistency. The CITC values of all items were >0.3, indicating that the data of each variable met the requirements of reliability.

The validity of the factors was then examined by exploratory factor analysis (EFA). The results of EFA are shown in Table 2. From Appendix 4 in Supplementary material, it can be seen that the Kaiser-Meyer-Olkin (KMO) value is 0.889, and the Bartlett's spherical test significance is <0.001, which is statistically significant. The findings in Table 2 show that the standardized factor loading values of the indicators ranged from 0.40 to 0.92, CR (Construct Reliability) values were above 0.7, and AVE (average variance extracted) values were above 0.5 (84). In summary, the convergent validity and discriminant validity are acceptable and can be tested with SEM.

**Table 2:** The results of factor analysis.

**Construct**	**Items**	**Factor loading**	**C.R**.	**AVE**
Attitude	AT1	0.88	0.74	0.51
	AT2	0.74		
	AT3	0.44		
Subjective norm	PGN1	0.44	0.90	0.52
	PGN2	0.40		
	PGN3	0.52		
	IN1	0.78		
	IN2	0.76		
	IN3	0.81		
	PGA1	0.83		
	PGA2	0.90		
	PGA3	0.84		
Perceived behavioral control	PBC1	0.81	0.87	0.63
	PBC2	0.82		
	PBC3	0.73		
	PBC4	0.81		
Gender difference	GD1	0.65	0.81	0.60
	GD2	0.83		
	GD3	0.89		
Peer academic ability	PAA1	0.88	0.86	0.67
	PAA2	0.86		
	PAA3	0.70		
Learning intention	LI1	0.85	0.91	0.77
	LI2	0.86		
	LI3	0.92		
Learning behavior	LB1	0.82	0.86	0.67
	LB2	0.78		
	LB3	0.85		

CR, construct reliability; AVE, average variance extracted.

### 5.3. Hypothesis testing

**Figure 4:**
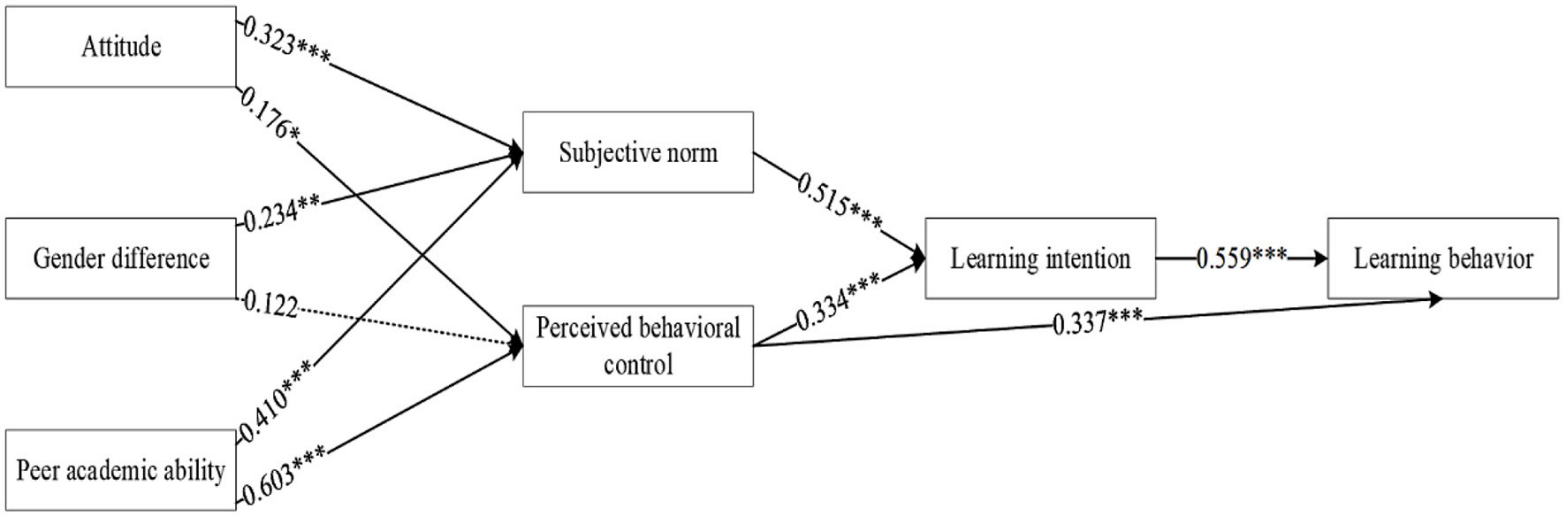
Overall results from the SEM. χ^2^/*df* = 4.664, CFI = 0.958, TLI = 0.906, SRMR = 0.049. *p < 0.05; **p < 0.01; ***p < 0.001.

The hypotheses were examined using MPLUS through the Bootstrap method and the results were represented in Figure 4. The specific coefficient results are shown in Table 3. The fit index of the structural equation model results was within the acceptable range, with χ^2^/*df* = 4.664 < 5, CFI = 0.958 > 0.90, and TLI = 0.906 > 0.90, SRMR (standardized root mean square residual) = 0.049 <0.08 (81, 85), indicating an adequate model fit.

**Table 3:** The results of examined effects using bootstrapping method.

**Significant effects**	**Estimate**	**SE**	**95% CI**
H1d: Attitude → Perceived behavioral control → Learning behavior	0.059^*^	0.032	(0.013, 0.087)
H1c: Attitude → Perceived behavioral control → Learning intention → Learning behavior	0.033^*^	0.015	(0.062, 0.229)
H4a: Peer academic ability → Subjective norm → Learning intention → Learning behavior	0.118^**^	0.034	(0.069, 0.224)
H4b: Peer academic ability → Perceived behavioral control → Learning behavior	0.203^**^	0.038	(0.120, 0.381)
H4c: Peer academic ability → Perceived behavioral control → Learning intention → Learning behavior	0.113^**^	0.038	(0.061, 0.234)
H5a: Gender difference → Subjective norm → Learning intention → Learning behavior	0.067^*^	0.027	(0.028, 0.147)

* p < 0.05;

** p < 0.01. Bootstrap = 5,000. CI, confidence interval.

In the results shown in Figure 4, attitude is positively related to subjective norm (β = 0.323, *p* < 0.001) and perceived behavioral control (β = 0.176, *p* < 0.05). Subjective norm (β = 0.515, *p* < 0.001) and perceived behavioral control (β = 0.334, *p* < 0.001) are positively related to learning intention. Perceived behavioral control (β = 0.337, *p* < 0.001) and learning intention (β = 0.559, *p* < 0.001) are positively related to learning behavior. Gender difference is positively related to subjective norm (β = 0.234, *p* < 0.01), but is not related to perceived behavioral control. So, H1a, H1b, H2, H3a, H3b and H6 are supported, H5b is rejected.

Next, we further tested the significance of mediating effects as shown in Table 3. The mediation effect was tested by estimating 95% confidence intervals (CI) for a randomly selected Bootstrap sample of 5,000, and if the confidence interval did not include 0, then the mediation effect was significant. As shown in the results, estimated value of path H1c is 0.059 [95% CI = (0.013, 0.087)], excluding 0, indicating that the mediating effect of perceived behavioral control is significant and H1c is verified. Estimated value of path H1d is 0.033 [95% CI = (0.062, 0.229)], excluding 0, indicating a significant multiple mediating effect of perceived behavioral control and learning intention, and H1d is verified. Estimated value of path H4a is 0.118 [95% CI = (0.069, 0.224)], excluding 0, indicating a significant multiple mediating effect of subjective norm and learning intention, and H4a is verified. Estimated value of path H4b is 0.203 [95% CI = (0.120, 0.381)], excluding 0, and H4b is verified. Estimated value of path H4c is 0.113 [95% CI = (0.061; 0.234)], excluding 0, and H4c is verified. Estimated value of path H5a is 0.067 [95% CI = (0.028, 0.147)], excluding 0, H5a is verified. The summary of the hypotheses testing results are shown in Table 4.

**Table 4:** Summary of hypotheses testing results.

**Hypothesis**	**Content**	**Result**
H1a	Attitude has a positive impact on subjective norms	Supported
H1b	Attitude has a positive impact on perceived behavioral control	Supported
H1c	Attitude positively influences engineering students' learning intentions through perceived behavioral control	Supported
H1d	Attitude positively influences engineering students' learning behavior through perceived behavioral control and learning intention	Supported
H2	Subjective norm has a positive impact on engineering students' learning intention	Supported
H3a	Perceived behavioral control has a positive effect on s engineering students' learning intention.	Supported
H3b	Perceived behavioral control has a positive effect on engineering students' learning behavior	Supported
H4a	Peer academic ability has an effect on learning behavior through subjective norm and learning intention	Supported
H4b	Peer academic ability positively influences learning behavior through perceived behavioral control	Supported
H4c	Peer academic ability positively influences learning behavior through perceived behavioral control and learning intention	Supported
H5a	Gender difference positively influences learning behavior through subjective norm and learning intention	Supported
H5b	Gender difference positively influences learning behavior through perceived behavioral control and learning intention	Not supported
H6	Learning intention has a positive effect on learning behavior	Supported

## 6. Discussion

The purpose of this empirical study was to test how the mechanism of peer pressure influenced the learning intentions of engineering students according to the theoretical lens of TPB. The study found that positive peer pressure can increase engineering students' learning intentions, which in turn drives learning behaviors, and emphasizes the importance of peer pressure in developing engineering students' learning behaviors. In addition, the results of the study also established the applicability of TPB and the extended model of TPB constructed in this study for investigating the role of peer pressure on learning behavior. The research has the following key findings:

1. The study found that attitudes based on TPB directly and positively influence subjective norms (*p* < 0.001) and perceived behavioral control (*p* < 0.05), with attitudes having a more significant effect on subjective norms. The pathway of the effect of attitudinal dimensions on engineering students' learning behavior through perceived behavioral control and willingness to learn was also identified. This result not only expands and reshapes the attitude relationship in the TPB model, but also identifies the mediating role of subjective norms and perceived behavioral control, and enhances the explanatory strength of the model. This also shows that positive attitudes influence learning intention and behavior in the context of peer pressure, which is consistent with the study by Azodo (48). Students' learning intentions can be judged or predicted from the strength of their attitudes toward learning, which plays an important role in influencing engineering students' learning behaviors. Specifically, a positive attitude toward learning can trigger positive peer pressure, and the more positive an engineering student's attitude toward learning is, the greater the degree of positive peer pressure he or she receives. When students believe that learning is a valuable and meaningful construct, they are more likely to take their professional courses seriously, and are willing to learn new and emerging skills in engineering fields that are constantly advancing. Then, students are subject to less negative peer pressure and more positive ones, and thus are willing to persist in their willingness to learn and thus engage in learning behaviors.
2. Subjective norms in peer pressure significantly influenced engineering students' learning intention (*p* < 0.001), compared to the direct effect of perceived behavioral control on intention. The result is consistent with the work of Huang et al. (56) and Veenstra et al. (86), suggesting that in a peer group with strict and orderly institutional norms, poor learning attitudes and behaviors will be curbed or punished by peers from external or psychological levels, thus stimulating the positive side of peer pressure to push students to learn. It is evident that the better the learning atmosphere of the peer group in which the engineering students are located, the greater the positive effect of peer pressure and the stronger the intention to learn. The reason for this may be that good group norms can induce peer pressure through rewards and punishments as a way to motivate students' willingness and behavior to learn. At the same time, students who slack off in their studies are ostracized and criticized by their peer group, which can have a strong impact on their self-esteem and inner feelings.
3. Peer pressure was found to positively influence engineering students' learning intention through the perceived behavioral control dimension (*p* < 0.001). This result is consistent with the findings of scholars such as Blackmore et al. (87) and Sarosa (88), indicating that engineering students' perceived ability is positively related to learning intention, i.e., the stronger the perception of stress, the stronger the willingness to learn. After analysis of the survey results, it was identified that most engineering students show high motivation to learn engineering skills and expertise with certainty, and when they felt peer pressure, students would mobilize positive learning emotions and believe that they could complete their tasks better and faster. In addition, the study also found that perceived behavioral control can directly and positively influence the learning behavior, which is consistent with the explanatory validity of Ajzen's (13, 89) model and further validates the standard TPB model.
4. In addition to the above three dimensions analyzed on the basis of TPB, the study also found that gender differences in peer pressure and peer academic ability have a positive effect on engineering students' learning behavior. Specifically, peer academic ability acts on learning behavior through subjective norm, perceived behavioral control and learning intention, and there are multiple chains of mediation paths. This result is consistent with scholars Moldes et al. (90) and Brouwer et al. (91) that when students are influenced and motivated by their peers who learn well, they obtain the support they need from their peer group, and focus more on their academic tasks. At the same time, imitation of peer behavior encourages students to discover new things outside their comfort zone; in turn, similar academic performance fosters friendships and helps them integrate into the class.
5. Gender differences in students influences the multiple chain mediated pathway of learning behavior through subjective norm and learning intention. Showing that gender differences influence the formation of group norms, consistent with the findings of scholars Ficano (4) and Hill (39), both male and female engineering students are also significantly motivated by high peer achievement; with high-performing female students motivating their male peers, thereby leading to higher graduation rates. In addition, the effect of gender difference on perceived behavioral control is not significant, which is probably because the traditional engineering education environment. This is generally regarded as a male-dominated classroom, lacking in suitable role models for women in academia and practice, and therefore there is a need to strengthen the training and attention paid to female engineering majors (92).

The findings have theoretical and practitioner related contributions for both providers of engineering education as well as engineering students. The theoretical implications are that this study applies TPB to the study of the effect of peer pressure on learning behavior, and determines that the TPB model can predict the learning behavior of engineering students under peer pressure, thereby extending the scope of application of TPB. The attitude relationship of TPB was extended and reshaped by identifying the role of direct positive influence of attitude on subjective norm and perceived behavioral control. The mediating role of subjective norm, perceived behavioral control and learning intention was also identified to enhance the explanatory strength of the model. In addition, understanding the forms and mechanisms of peer pressure effects are of theoretical prominence for evaluating engineering students' peer interaction skills and their smooth adaptation to group life in order to improve academic performance.

This findings have practical implications for the use of appropriate peer pressure by engineering educators, especially the use of reward and penalty-oriented structures to improve engineering students' academic performance as well as overall competencies, effectively contributing to the development of engineering talent employability. The study also enables engineering students to better understand and adapt to peer pressure in their university studies and life, and transform their studies into an effective learning aid that helps them achieve their longer-term goals such as academic and career development, and better integrate into the trends of the digital age.

## 7. Conclusion

This study explored the role of engineering students' peer pressure on learning behavior using the theory of planned behavior (TPB) as a core theoretical framework. Based on TPB, three peer pressure dimensions of engineering students' attitudes, subjective norm, and perceived behavioral control, as well as two new variables of gender differences and peer academic ability, were generalized through reviewing the literature. Finally, a new TPB extension model (PP-LB impact mechanism model) was established, and a questionnaire was designed on this basis, whereby SEM was used as the test method. The result showed that positive peer pressure can increase engineering students' learning intention and thus promote learning behavior. It was also determined that the TPB model can effectively explain the effect of peer pressure on learning behavior, in addition to expanding and reshaping the relationship between the attitudinal dimension in the TPB model, i.e., attitude can positively influence perceived behavioral control and subjective norms.

Based on the results of the study, the following suggestions are provided.

1. Peer pressure can encourage students to make progress together. Engineering educators should make reasonable use of peer pressure in student groups to guide engineering students to change their learning attitudes and stimulate the positive side of peer pressure. For example, teachers should continuously clarify to students during their freshman and sophomore years the benefits of their technical knowledge in relation to their future career development in the digital era, so that students can change their learning attitudes. Students should also be educated about the importance of peer pressure on learning so that they can turn their anxiety into motivation in the face of pressure.
2. Engineering educators should use more reward and less penalty-oriented structures to motivate engineering students to learn. For example, group competitions are appropriate in engineering classrooms as they are a powerful external motivator and can stimulate positive peer pressure when students' self-esteem and sense of honor are combined. Educators also can use a team-based learning model in the classroom to strengthen connections between groups of students, where active classroom interactions help build academic networks. This approach can support an improvement for peers to encourage each other in their studies, engage in reasonable competition, and reinforce individual self-directed learning, which can be beneficial to academic performance.
3. Engineering educators should acknowledge gender differences in peer pressure and the influence of academic level to rationalize the study groups or teams of engineering students. For example, collaborative learning groups should include students with higher and lower academic levels, and each group should contain male and female students as a way to learn from and motivate each other.

## 8. Limitations and future study

The limitations of this study are that, firstly, the participants of the research survey were mainly engineering students. In future studies, interviews can be conducted with engineering teachers to understand the effect of peer pressure on engineering students' learning, and to expand the sample size of the study and further test the hypothesis model to determine the causality of the study. Secondly, students' learning intentions may be provisional, meaning that some of the students' learning intentions presented in the research study may be based on present-day intentions, and individual behavioral intentions may change as they develop over time. In addition, self-assessment was used in the study to measure learning behaviors. Although the applicability of the scale has been verified and the use of the self-assessment method is based on the ability of individuals to evaluate themselves accurately, individuals may also overestimate their own intentions. Therefore, more studies are needed on the actual learning behaviors of engineering students, and follow-up studies should focus on changes in learning intentions and behavior over time. A more rigorous study design may also be adopted to continuously track and monitor engineering students' behaviors and conduct research covering the entire behavioral cycle.

## Data availability statement

The raw data supporting the conclusions of this article will be made available by the authors, without undue reservation.

## Author contributions

JZha contributed to the conception of the study. LX and YD performed the experiment, data analyses, and wrote the manuscript. JZha and JZhe contributed significantly to analysis and manuscript preparation. GS and WZ helped perform the analysis with constructive discussions. SP contributed to the revision and various enhancements of the article content. All authors contributed to the article and approved the submitted version.
