# Erratum: Understanding the role of peer pressure on engineering students' learning behavior: a TPB perspective

**DOI:** 10.3389/fpubh.2023.1297534

**Published:** 2023-09-27

**Authors:** Frontiers Production Office

**Affiliations:** Frontiers Media SA, Lausanne, Switzerland

**Keywords:** peer pressure, peer influence, theory of planned behavior, engineering students, learning intention, learning behavior

Due to a production error, affiliations 4 and 5 were in the incorrect order. The correct affiliation list is: ^1^School of Foreign Languages, Northwest University, Xi'an, China, ^2^School of Economics and Management, Chang'an University, Xi'an, China, ^3^Faculty of Civil Engineering and Mechanics, Kunming University of Science and Technology, Kunming, China, ^4^School of Civil Engineering, Zhengzhou University, Zhengzhou, Henan, China, ^5^Institute of China's Science Technology and Education Policy, Zhejiang University, Hangzhou, China, ^6^School of Engineering, London South Bank University, London, United Kingdom

The publisher apologizes for this mistake.

The original article has been updated.

