# Supplementary material for: Understanding the role of peer pressure on engineering students' learning behavior: A TPB perspective
Source: Front Public Health. 2023 Jan 6;10:1069384. doi: 10.3389/fpubh.2022.1069384 (PMC9852900; doi:10.3389/fpubh.2022.1069384)
Supplement: Supplementary file 1 [file Table_1.docx]

## Appendix

## Appendix 1：

The scale of peer pressure on engineering students’ learning intention

| Variable | Item  labels | Items | Source |
| --- | --- | --- | --- |
| Attitude | AT1 | I will take my professional skills and courses seriously. | (105)  (106, 107) |
|  | AT2 | I am willing to try to learn different fields of knowledge. |  |
|  | AT3 | Peer pressure can motivate me to learn. |  |
| Peer Group Norms | PGN1 | I care about what others think of my performance in my studies. | (107, 108) |
|  | PGN2 | Being able to win the recognition and appreciation of others is what drives me to study hard. |  |
|  | PGN3 | In class discussions, I care about my peers' reactions to the ideas I express. |  |
| Peer Group Atmosphere | PGA1 | When my peer group is committed to learning and doing well, I feel a sense of urgency to learn and try to engage in my studies. | (109, 110) |
|  | PGA2 | A good peer learning atmosphere makes my learning more effective. |  |
|  | PGA3 | A positive group atmosphere of cooperation will also inspire me to participate. |  |
| Inner Feeling | IN1 | In teamwork learning, I feel guilty if I don't complete my tasks. | (111, 112) |
|  | IN2 | I would be ashamed to be at the bottom of the peer group in terms of grades. |  |
|  | IN3 | I would be lost because my academic achievements were not as good as those of my peers and would want to make a difference. |  |
| Perceived behavioral control | PBC1 | When facing difficult tasks, I am certain that I will accomplish them. | (107, 113, 114) |
|  | PBC2 | I can make judgments about the learning difficulty of a target course or skill. |  |
|  | PBC3 | I am highly motivated to learn professional knowledge or skills that I am sure of. |  |
|  | PBC4 | When I feel peer pressure, I am better and faster at completing set tasks. |  |
| Gender difference | GD1 | The learning outcomes of my peers of the opposite gender can stimulate my interest in learning. | (41, 43, 115) |
|  | GD2 | I am able to communicate more smoothly with my same-gender peers in terms of collaboration in learning. |  |
|  | GD3 | The high academic achievement of my same-gender peers can motivate me to learn. |  |
| Peer academic ability | PAA1 | When I see the achievements of my peers, I motivate myself to make progress. | (27, 115) |
|  | PAA2 | When interacting with students at different levels, I am willing to learn from their strengths. |  |
|  | PAA3 | I will start learning this technology because my peers have mastered the emerging engineering technology. |  |
| Learning intention | LI1 | Willing to communicate with peers and learn from each other. | (116, 117) |
|  | LI2 | Willingness to invest time and effort to improve learning and academic performance. |  |
|  | LI3 | Willingness to upgrade their soft and hard skills to improve engineering employability. |  |
| Learning behavior | LB1 | I am actively involved in team learning activities in the classroom. | (118, 119) |
|  | LB2 | I am constantly learning new engineering expertise and new technologies. |  |
|  | LB3 | I will take the initiative to make time to learn. |  |

## Appendix 2:

Descriptive statistics and correlation analysis

The descriptive statistics and correlation of studied variables

|  | Mean | S.D. | 1 | 2 | 3 | 4 | 5 | 6 | 7 |
| --- | --- | --- | --- | --- | --- | --- | --- | --- | --- |
| 1. Attitude | 4.265 | 0.614 | (0.830) |  |  |  |  |  |  |
| 2. Subjective norm | 4.094 | 0.633 | 0.679^***^ | (0.807) |  |  |  |  |  |
| 3.Perceived behavioral control | 3.958 | 0.714 | 0.597^***^ | 0.650^***^ | (0.844) |  |  |  |  |
| 4. Gender difference | 3.777 | 0.759 | 0.464^***^ | 0.657^***^ | 0.605^***^ | (0.840) |  |  |  |
| 5. Peer academic ability | 4.056 | 0.720 | 0.604^***^ | 0.761^***^ | 0.791^***^ | 0.666^***^ | (0.863) |  |  |
| 6. Learning intention | 4.181 | 0.700 | 0.567^***^ | 0.733^***^ | 0.670^***^ | 0.624^***^ | 0.760^***^ | (0.922) |  |
| 7. Learning behavior | 3.950 | 0.797 | 0.537^***^ | 0.721^***^ | 0.712^***^ | 0.647^***^ | 0.759^***^ | 0.785^***^ | (0.894) |

Note: The values in the diagonal parathesis are the square root of average variance extracted (AVE).  ^***^ *p* < 0.001. SD=standard deviation.

## Appendix 3:

Results of CITC and alpha-coefficient analysis of study variables

| Dimension | Variable | Corrected Item and Total  Correlation | Alpha | |
| --- | --- | --- | --- | --- |
| Attitude | AT1 | 0.494 | 0.764 | |
|  | AT2 | 0.428 |  |  |
|  | AT3 | 0.563 |  |  |
| Subjective norm | PGN1 | 0.469 | 0.898 | 0.911 |
|  | PGN2 | 0.418 |  |  |
|  | PGN3 | 0.526 |  |  |
|  | PGA1 | 0.763 | 0.843 |  |
|  | PGA2 | 0.806 |  |  |
|  | PGA3 | 0.702 |  |  |
|  | IN1 | 0.714 | 0.852 |  |
|  | IN2 | 0.72 |  |  |
|  | IN3 | 0.743 |  |  |
| Perceived behavioral control | PBC1 | 0.634 | 0.863 | |
|  | PBC2 | 0.679 |  |  |
|  | PBC3 | 0.663 |  |  |
|  | PBC4 | 0.752 |  |  |
| Gender difference | GD1 | 0.586 | 0.770 | |
|  | GD2 | 0.655 |  |  |
|  | GD3 | 0.705 |  |  |
| Peer academic ability | PAA1 | 0.815 | 0.845 | |
|  | PAA2 | 0.77 |  |  |
|  | PAA3 | 0.674 |  |  |
| Learning intention | LI1 | 0.837 | 0.913 | |
|  | LI2 | 0.713 |  |  |
|  | LI3 | 0.735 |  |  |
| Learning behavior | LB1 | 0.786 | 0.871 | |
|  | LB2 | 0.67 |  |  |
|  | LB3 | 0.704 |  |  |

Note: CITC=Corrected Item-Total Correlation

## Appendix 4:

| KMO and Bartlett’s test | | |
| --- | --- | --- |
| KMO |  | 0.889 |
| Bartlett test of sphericity | Approx. Chi-square | 2571.053 |
|  | df | 406 |
|  | Sig. | 0.000 |

Note: KMO= Kaiser-Meyer-Olkin
